# Flowering phenology differs among wet and dry sub-alpine meadows in southwestern China

**DOI:** 10.1093/aobpla/plae002

**Published:** 2024-01-18

**Authors:** Shristhi Nepal, Judith Trunschke, Zong-Xin Ren, Kevin S Burgess, Hong Wang

**Affiliations:** Key Laboratory for Plant Diversity and Biogeography of East Asia, Kunming Institute of Botany, Chinese Academy of Sciences, 132 Lanhei Road, Kunming 650201, China; University of Chinese Academy of Sciences, No.1 Yanqihu East Rd, Huairou District, Beijing 101408, China; Key Laboratory for Plant Diversity and Biogeography of East Asia, Kunming Institute of Botany, Chinese Academy of Sciences, 132 Lanhei Road, Kunming 650201, China; Nature Conservation and Landscape Ecology, Faculty of Environment and Natural Resources, University of Freiburg, Tennenbacher Str., 479106 Freiburg, Germany; Key Laboratory for Plant Diversity and Biogeography of East Asia, Kunming Institute of Botany, Chinese Academy of Sciences, 132 Lanhei Road, Kunming 650201, China; Department of Biomedical Sciences, Mercer University School of Medicine, Columbus, GA 31901, USA; Key Laboratory for Plant Diversity and Biogeography of East Asia, Kunming Institute of Botany, Chinese Academy of Sciences, 132 Lanhei Road, Kunming 650201, China

**Keywords:** floral longevity, floral rewards, flower, flowering duration, phenology, pollen number

## Abstract

The effect of floral traits, floral rewards and plant water availability on plant–pollinator interactions are well-documented; however, empirical evidence of their impact on flowering phenology in high-elevation meadows remains scarce. In this study, we assessed three levels of flowering phenology, i.e. population-, individual- and flower-level (floral longevity), in two nearby but contrasting (wet versus dry) sub-alpine meadows on Yulong Snow Mountain, southwestern China. We also measured a series of floral traits (pollen number, ovule number, and the ratio of pollen to ovule number per flower, i.e. pollen:ovule ratio [P/O]) and floral rewards (nectar availability and pollen presentation) as plausible additional sources of variation for each phenological level. Floral longevity in the wet meadow was significantly longer than that for the dry meadow, whereas population- and individual-flowering duration were significantly shorter. Our results showed a significant positive relationship between flowering phenology with pollen number and P/O per flower; there was no relationship with ovule number per flower. Further, we found a significant effect of flowering phenology on nectar availability and pollen presentation. Our findings suggest that shorter floral longevity in dry habitats compared to wet might be due to water-dependent maintenance costs of flowers, where the population- and individual-level flowering phenology may be less affected by habitats. Our study shows how different levels of flowering phenology underscore the plausible effects of contrasting habitats on reproductive success.

## Teaser

Flowering phenology differs among wet and dry sub-alpine meadows on Yulong Snow Mountain, southwestern Yunnan. There is a significantly shorter duration of floral longevity in dry meadow compared to floral longevity in wet meadow, suggesting the water-dependent maintenance costs of flowers may play a role for flowering phenology of sub-alpine plant species.

## Introduction

Flowering phenology, the timing of flowering events, is an important component of a flowering plant’s reproductive success and survival. Patterns of flowering phenology can vary among different flowering populations of a species within a community (population-level), individuals within a population (individual-level) and even among flowers within an individual (flower-level, i.e. floral longevity) (Bosch *et al.*, 1997; Forrest & Miller-Rushing, 2010; Shivanna & Tandon, 2014; Song *et al.*, 2022). For example, at the population-level, some species can produce many flowers or flowering individuals over a short period (i.e. mass flowering) or can produce few flowers/individual over a longer duration (i.e. steady-state) ( Haggerty and Mazer, 2008; Oleques *et al.*, 2017). Patterns of flowering phenology can represent the gradient of flowering strategies determined by the intensity and synchrony of flowering at each phenological level (i.e. population-, individual-, and flower-level) (Koptur *et al.*, 1988; Oleques *et al.*, 2017). Additionally, these patterns within the plant community can be influenced by the costs of flowering/reproductive maintenance (e.g. pollen number, ovule number, P/O per flower, floral rewards; Bosch *et al.*, 1997; Fabbro and Körner, 2004; Morales *et al.*, 2005; Ansquer *et al.*, 2009), as well as environmental factors (i.e. temperature and soil moisture; Kudo and Hirao, 2006; Arroyo *et al.*, 2013; Cortés-Flores *et al.*, 2017; Jnawali and Neupane, 2021). Investigating sources of variation due to floral traits and environmental factors on flowering phenology may help to discern the complex factors associated with the impacts of drought conditions on insect-pollinated flowering plant species in response to future drought conditions.

Population-level flowering phenology, population flowering duration (population FD), is quantified as the overall duration of flowering events from when the first flower opens to the last flower in the wilting stage in a specific habitat (Price and Waser, 1998; Haggerty and Mazer, 2008; Caradonna *et al.*, 2014; Shivanna and Tandon, 2014). The population FD of a species within a community can influence species abundance and their temporal presence or absence in an ecosystem and, as such, can serve as an indicator of community assembly (Rathcke and Lacey, 1985; Haggerty and Mazer, 2008). In most communities, flowering time overlaps between species, generating the potential for competition among pollinators and facilitating pollinator resources throughout the entire flowering season (Sargent and Ackerly, 2008; Crimmins *et al.*, 2011; Gallagher and Campbell, 2020). Given that community structure regulates the types and magnitude of interactions among the flowering individuals and species within its habitat (Heinrich, 1976; Elzinga *et al.*, 2007; Catorci *et al.*, 2012), population FD may, in turn, be a key factor in structuring plant communities, even though we know little about its variation in natural communities.

Individual plants within a species may also vary significantly in their flowering duration. The flowering duration of an individual plant (individual FD) includes the time an individual has its first open functional flower to the last flower in the wilting stage (Craine *et al.*, 2012; Shivanna and Tandon, 2014). Individual FD can strongly impact a plant’s reproductive success by determining whether the flowers produced are pollinated and if seed production, dispersion, and germination successfully occur (Rathche and Lacey, 1985; Ramírez *et al.*, 1998; Shivanna and Tandon, 2014). Several studies have shown that in addition to individual FD, selection for additional phenological response variables is likely influenced by genetic and environmental factors, including the onset of flowering and the average number of flowers per individual blooming each day during the flowering season (Blionis *et al.*, 2001; Dunne *et al.*, 2003). Relatively few studies, however, have thoroughly explored the environmental factors, particularly those related to wet and dry habitats, driving natural variation in individual FD.

Flowering phenology can also be examined at the level of a flower within an individual plant (floral longevity). Here, floral longevity represents the period from when a flower first opens and is functional until it wilts (Primack, 1985; Arroyo *et al.*, 2013; Wang *et al.*, 2018). Floral longevity is a key reproductive trait in animal-pollinated species that determines the chances of pollen dispersal and pollen capture by stigmas (Primack, 1985; Ashman and Schoen, 1994; Ashman *et al.*, 2004; Miller-Rushing and Primack, 2008; Song *et al.*, 2022). Furthermore, it is also associated with the mating system (Primack, 1985; Ashman *et al.*, 2004; Lozada-Gobilard *et al.*, 2019), the cost of flower production and floral rewards (Stratton, 1989; Ashman and Schoen, 1994; Gao *et al.*, 2015; Descamps *et al.*, 2020, 2021; Pyke and Ren, 2023), and as such, can influence the number and quality of offspring produced (Ashman and Schoen, 1994; Evanhoe and Galloway 2002; Itagaki and Sakai 2006; Song *et al.*, 2022). Although determining the magnitude of floral longevity variation across contrasting habitats (e.g. wet versus dry) could be key to understanding how flowering phenology varies among species and individuals, a paucity of studies empirically test this potentially significant source of variation.

Abiotic factors, those associated with the surrounding ecological environment, i.e. temperature and water availability, can influence the cost of flower maintenance through respiration and transpiration, directly affecting flowering phenology (Primack, 1985; Yasaka *et al.*, 1998; Ashman *et al.*, 2004; Pacheco *et al.*, 2016; Pyke and Ren, 2023). For example, drier habitats can influence those floral characteristics significantly associated with pollination, fertilization and viable seed production. Such key floral traits, i.e. flowering duration, floral male and female gamete production, and floral rewards, can have a significant impact on pollinator as well as reproductive output and ultimately impacting future plant establishment, persistence, and community structure (Haggerty and Mazer, 2008; Miller-Rushing and Inouye, 2009; Descamps *et al.* 2021; Kuppler *et al.*, 2021; Höfer *et al.*, 2022). Therefore, we predict that such factors are also likely to impact population- and individual FD negatively, resulting in a significant decrease in offspring number and quality in the overall community (Jorgensen and Arathi, 2013; Kehrberger and Holzschuh, 2019; Cunha *et al.* 2022; Song *et al.*, 2022).

Understanding how various levels of flowering phenology respond to different environmental habitats is an essential aspect of plant reproduction that is rarely investigated. In this study, we aim to investigate patterns of flowering phenology between a wet and a dry sub-alpine meadow on Yulong Mountain, Hengduan Mountains region, southwestern China. Specifically, we examined how three levels of phenology (i.e. population-, individual- and flower-level) varied in response to habitat type (wet versus dry), floral traits, and floral rewards. We addressed the following questions: (i) Do patterns of flowering phenology differ between the wet and dry habitats? (ii) Does variation in pollen number, ovule number and P/O per flower explain the distribution of flowering phenology between a wet and dry habitat? (iii) Is there a relationship between flowering phenology and floral rewards, i.e. nectar availability and pollen presentation?

## Materials and Methods

### Study area

A field study was conducted on Yulong Snow Mountain (27°00’N, 100°10’E), Hengduan Mountains region, southwestern China, during the flowering season (early-June to mid-September) 2021. The mountain hosts a large diversity of flowering plants with more than 2,815 species (Liu *et al.* 2015), including different sub-alpine and alpine meadow habitats. The dominant vegetation type of the mountain is *Pinus yunnanensis* forest at lower elevation, *Abies*-*Rhododendron* forest at mid-elevation, and dwarf *Rhododendron* forest at higher elevation. The study sites were located on the mid-sub-alpine elevation belt, where wet and dry meadows were located. The study sites were located at the Lijiang Forest Biodiversity National Observation and Research Station (27°00’09‘ N, 100°10’57’ E) on Yulong Snow Mountain, Yunnan, China.

**Figure 1.**
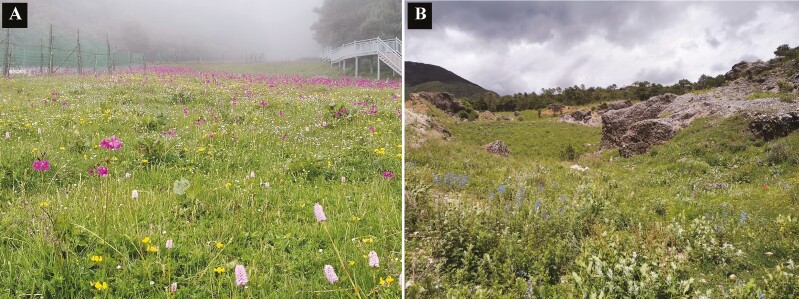
The study sites at the Lijiang Forest Biodiversity National Observation and Research Station, Yulong Mt., southwestern China. View of the (A) wet meadow and (B) dry meadow.

We selected two natural meadows in the sub-alpine community, one in a wet and the other in a dry habitat, approximately 500 m apart at the same elevation (3200 m a.s.l., Fig. 1). The wet meadow was near a water reservoir with a stream running through the meadow, whereas the dry meadow was a dry grassland along a dirt track in close proximity. The wet meadow was intensely used for pollination and floral traits studies (Zhao *et al.*, 2019; Xu *et al.*, 2021; Nepal *et al.*, 2023). In both meadows, we included all insect-pollinated flowering plant species, comprising 21 species in the wet habitat and 20 species in the dry habitat, excluding wind-pollinated ones, e.g. some members of Poaceae and Juncaceae. Our study covers almost the entire flowering period (4 months) in both meadows from the beginning of June to mid-September.

### Flowering phenology

#### Population flowering duration

To determine population FD, we laid one 100-m-long transect across each meadow. To avoid edge effects, we set up transects within the centre of each meadow. From early-June to mid-September 2021, we recorded all the plants with open flowers within 100 (1 × 1 m) quadrates continuously along the transect once every ten days, in total 12 times in the wet-meadow and 11 times in the dry-meadow; the dry-meadow started flowering a week after we started fieldwork. Specifically, within each quadrate, we recorded the number of flowering individuals of each species and the number of fully opened flowers for each flowering individual. Within our wet-meadow transect, we monitored 17 species with 5,890 flowering individuals (19,652 open flowers) and 20 species with 4,401 (26,982 open flowers) in the dry meadow. The overall flowering duration of each species in each meadow was estimated by calculating when a species within the transect has its first to last open flower. Species and floral abundance in each meadow were measured as the number of flowering individuals and the number of open flowers during each visit, respectively. During the collection of population FD data, four species (*Arenaria barbata*, *Parnassia mysorensis*, *Pedicularis densispica*, *Pe*. *gruina*) did not occur within our transect and were considered unavailable data for population FD analysis; however, flowering individuals of those species were present in the meadow and were collected for all other data analysis.

Further, *Halenia elliptica* and *Gentiana pubigera* finished flowering after we completed our survey and were, therefore, not included in the population FD analysis. As three species (*Lotus corniculatus*, *Polygonum nepalensis*, and *Potentilla lancinata*) co-occur in the two meadows, we first collected population FD data for all three species in both meadows along the transects. Among these species, we observed many flowering individuals of *Lo. corniculatus*, *Pol. nepalensis* in the wet meadow and *Pot. lancinata* in the dry meadow. Therefore, we selected population FD data for these three species only from that meadow where their abundance was notably higher.

#### Individual flowering duration

To estimate the length and pattern of individual FD, we randomly selected and marked 30 bolting individuals for each species (21 species in wet-meadow and 20 species in dry-meadow). We tagged 1,230 individuals of 41 species according to their flower open time throughout the flowering seasons in both communities. The tagged individuals were visited every 2–3 days until the last flower was wilted on each respective individual. We counted the number of open flowers per species, and individual FD was calculated as the number of days from when an individual had its first open flower to when its last flower wilted. The first flowering date was defined as the first day an individual plant had its first open flower, and the last flowering date was the day on which the individual had its last flower in a wilting stage. The first and last flowering dates were used to estimate the overall individual FD of each individual. We then calculated the peak flowering date for each individual as the day on which 50% of that individual’s flowers had already opened. Similarly, the duration of peak flowering was estimated as the days on which 30–70% of an individual’s flowers were opened (CaraDonna *et al.*, 2014).

#### Floral longevity

We randomly marked three flower buds from 25 to 30 individuals to quantify the longevity of individual flowers for each species (21 species in the wet meadow and 20 species in the dry meadow). We marked 3,690 flower buds for 41 species throughout the flowering seasons. In the case of species with aggregated small florets that make a single flower head (e.g. flowers of Asteraceae, Polygonaceae), we marked one flower head and recorded when it has anthers available to the pollinators to the wilting stage, and the overall duration was taken as floral longevity. All the marked flower buds were visited daily; we recorded the date the bud opened and the date the flower wilted. The flower longevity was considered the days from the flower opening date to the flower wilting date.

### Quantification of floral traits and floral rewards

To quantify the floral traits (i.e. pollen and ovule number per flower), we collected 10 matured flower buds from different individuals (one bud per individual) of each species (21 species in the wet meadow and 20 species in the dry meadow) from both communities. We used a light microscope with a haemocytometer to count pollen numbers and a stereo-microscope to count ovule numbers. To estimate the pollen number per anther, we prepared a pollen suspension of one anther (1 mL). We used a known volume of distilled water (20 µL) to count all pollen grains in the hemocytometer. This count was then used to calculate the number of pollen grains in a single anther. Further, the number of pollen grains in a single flower was calculated by multiplying the number of pollen grains in one anther by the number of anthers in one flower (see details in Nepal *et al.*, 2023). In species of Asteraceae, we considered all the pollen and ovules present within one flower head as the number of pollen and ovules per flower following (Arroyo *et al.*, 2019; Nepal *et al.*, 2023). To estimate the number of ovules per flower, we dissected the ovary and counted all the ovules under the stereo-microscope (Kearns *et al.*, 1998). We calculated P/O by dividing the number of pollen grains in each flower by the number of ovules in the same flower.

We evaluated floral rewards (nectar availability and pollen presentation) directly in the field by observing 1–3 fresh open flowers on ten plants of each species (21 species in the wet meadow and 20 species in the dry meadow). To determine nectar availability, we examined flowers and noted the presence or absence of a watery substance at the base of the floral tube and the base of the style/ovary to determine nectar availability in a flower (i.e. absence versus presence). We determined pollen presentation by examining whether the pollen in a flower was readily available for pollinators or protected within the flower structure (i.e. pollen open-available versus pollen enclosed).

### Statistical analysis

The overall data regarding each variable were averaged to 41 species (except for population FD, *n* = 37 species) and then used for further analysis. We conducted the Shapiro–Wilks Normality test to determine the normality of the data, resulting in a significant right-skewness in the data (Shapiro–Wilks test, *P* < 0.001). We used a generalized linear model (GLM, with Poisson distribution) to show the differences in each level of flowering phenology across wet and dry habitats. To estimate the relationship of each level of flowering phenology with pollen number, ovule number, and P/O per flower, respectively, we performed GLM regression with Quasi Poisson distribution; the data were over-dispersed across combined as well as wet and dry meadows. Further, to estimate the effects of nectar availability and pollen presentation on each of the three respective levels of flowering phenology across both meadows, we performed multiple regression GLM with a Poisson distribution, given the data regarding each level of floral longevity is count data. All the analyses were done using R version 4.0.2 (R Core Team 2019).

## Results

### Flowering phenology

**Figure 2.**
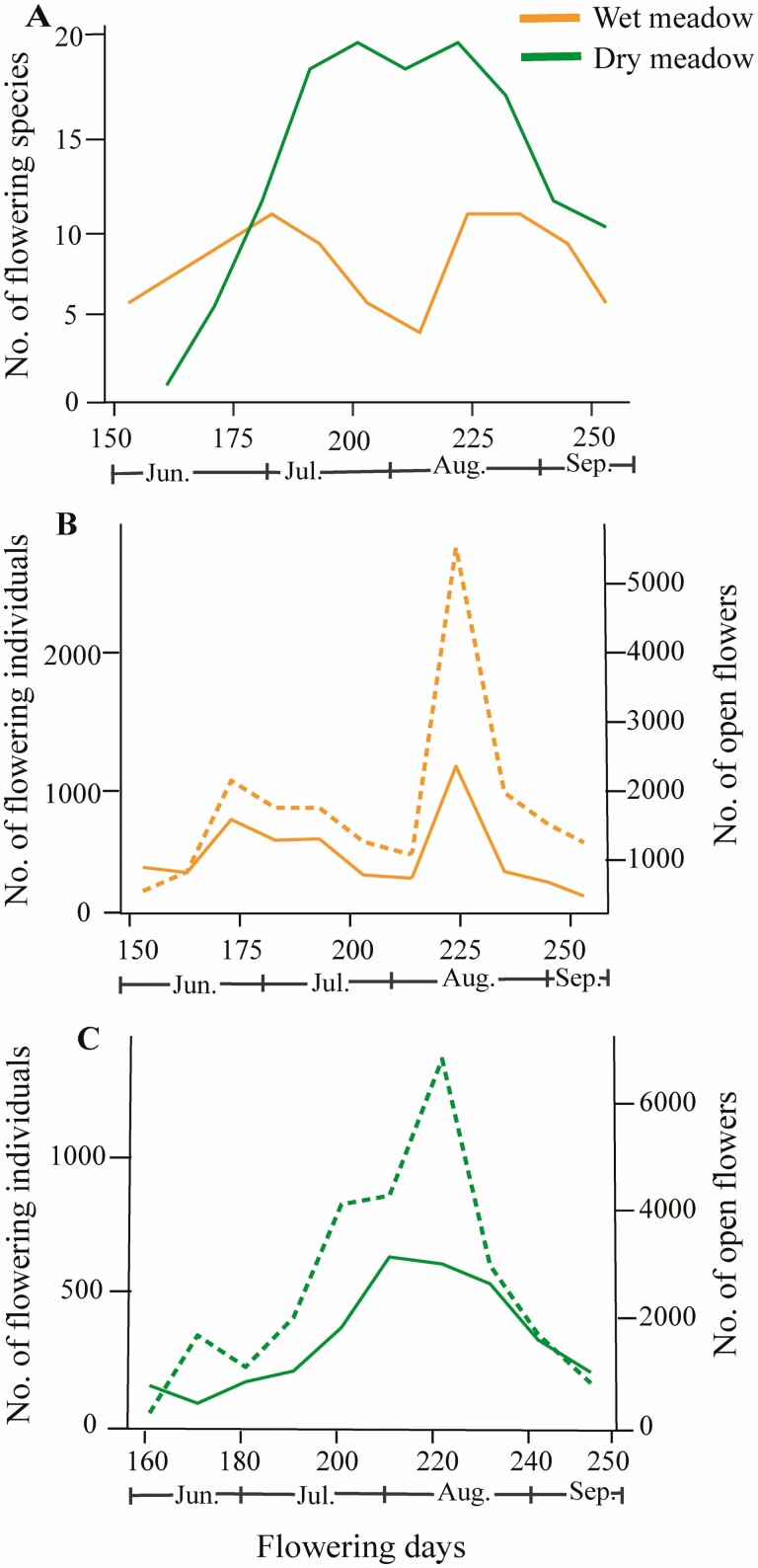
Flowering phenology across two meadows on Yulong Mt., southwestern China. (A) Number of flowering species in the wet meadow (orange solid line) and dry meadow (green solid line). In panels B and C, the solid line represents the number of flowering individuals on the left vertical axis, and the dashed line represents the number of open flowers on the right vertical axis. The colour in the graph represents the habitat, i.e. the orange line (solid and dashed) indicates wet meadow, and the green line (solid and dashed) indicates dry meadow. Sampling days are shown in the horizontal bar as the day of the year. *N* = 41.

**Figure 3.**
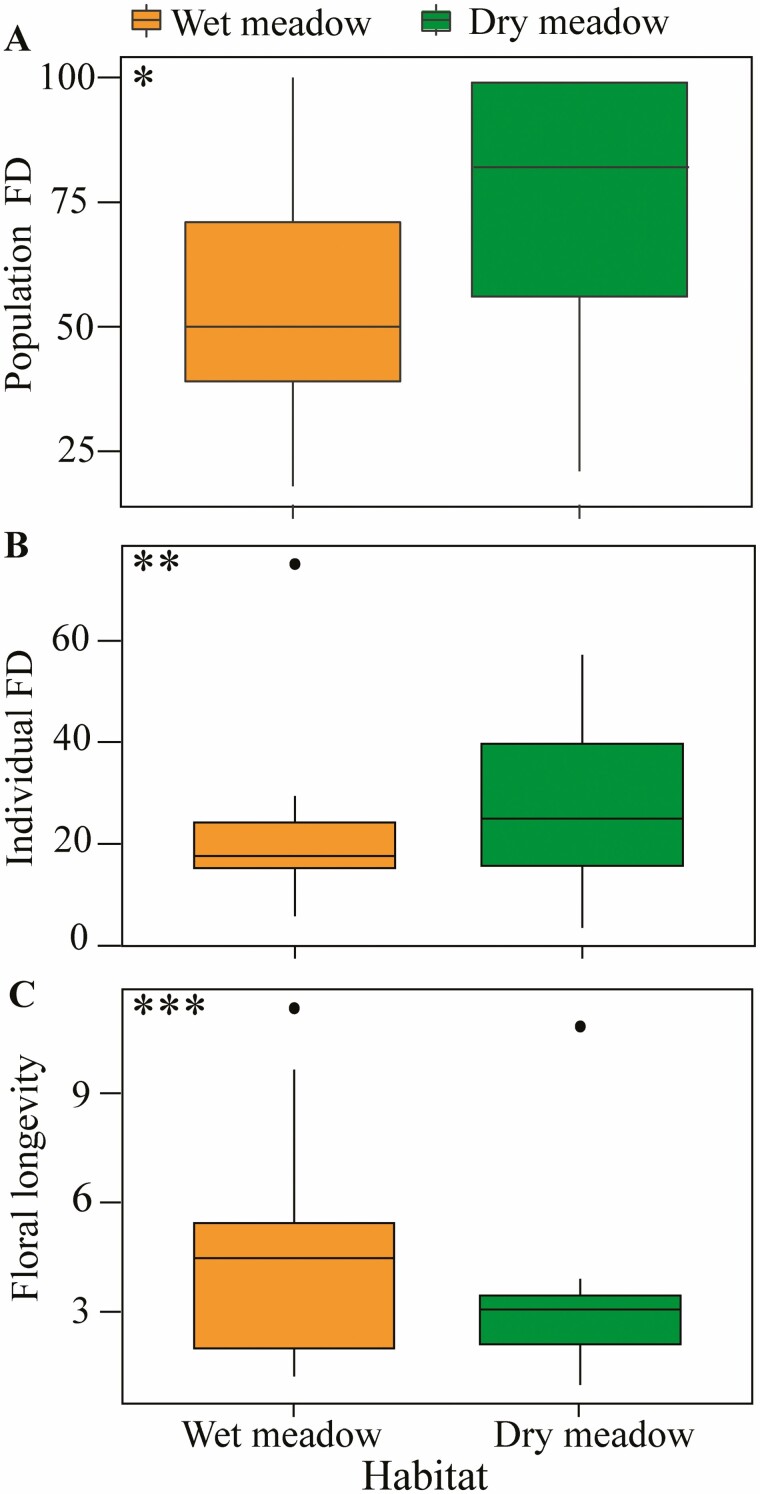
Flowering phenology in the wet and dry meadow. (A) Mean population FD. (B) Mean individual FD. (C) Mean floral longevity on Yulong Mt., southwestern China. Box plots with orange representing wet-meadow and green representing dry-meadow. 25th and 75th percentiles represent lower and upper box boundaries, respectively; the horizontal line inside the box represents the median. The 90th percentile is represented by the upper error lines with filled circles with data falling outside. Significant differences are based on the Kruskal–Wallis non-parametric ANOVA: *P* < 0.05*; *P* < 0.01**; and *P* < 0.001***. *N* = 37 (population FD) and 41 (individual FD and floral longevity).

The seasonal distribution of the number of flowering species, flowering individuals and open flowers differed between wet and dry meadows (Fig. 2). In the wet meadow, we found two peaks for the number of flowering species in bloom, the number of flowering individuals and the number of open flowers: one during late June/early July and a second in mid-August (Fig. 2A and B). In contrast, we found a near hump-shaped pattern with the number of flowering species, flowering individuals and open flowers with only one blooming peak during late July to mid-August in the dry meadow (Fig. 2A and C). The mean population FD and individual FD were significantly shorter in the wet meadow (52.47 ± 5.83 and 21.08 ± 3.04, respectively) compared with dry meadows (57.54 ± 7.39 and 28.16 ± 3.59, respectively; Fig. 3A and B), whereas mean floral longevity was significantly longer in the wet meadow (3.12 ± 0.44, *P* < 0.001) than in the dry meadow (4.63 ± 0.65) (Fig. 3C).

#### Population flowering duration (population FD)

**Figure 4:**
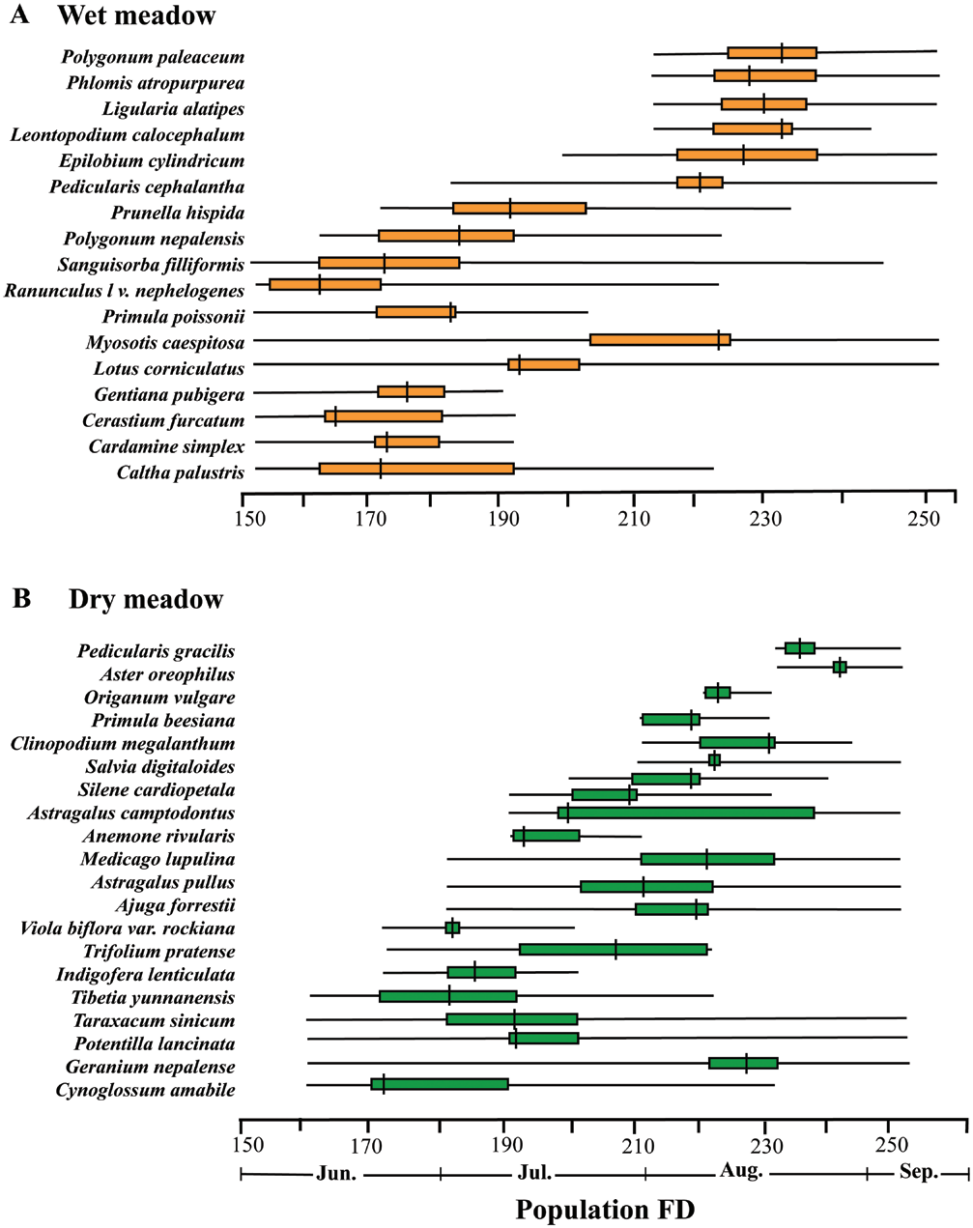
Population FD (population flowering duration among species) in two contrasting sub-alpine meadows (wet versus dry) on Yulong Mt., southwestern China. Shown are FD for a (A) wet and (B) dry meadow. The solid thin line represents the entire flowering period of each species; the coloured bars indicate the peak flowering period (30–70% of flowers opened), and the vertical bar indicates the peak flowering day (50% of flowers opened). *N* = 37.

Population FD varied widely across the entire community, ranging from 18 days (*Saxifraga diversifolia*) to 100 days in *Myosotis caespitosa* (Fig. 4A and B). In the wet meadow, *M. caespitosa* and *Lo. corniculatus* showed the longest FD (100 and 96 days, respectively), whereas *Gen. pubigera* and *Leontopodium calocephalum* had the shortest FD (25 and 31 days, respectively). In the dry meadow, *Taraxacum sinicum*, *Pot. lancinata* showed the longest FD (92 days for each species). In contrast, the shortest FD was in *Aster oreophilus* and *Viola biflora* var*. khasiana* (21 and 30 days, respectively).

#### Individual flowering duration (individual FD)

**Figure 5.**
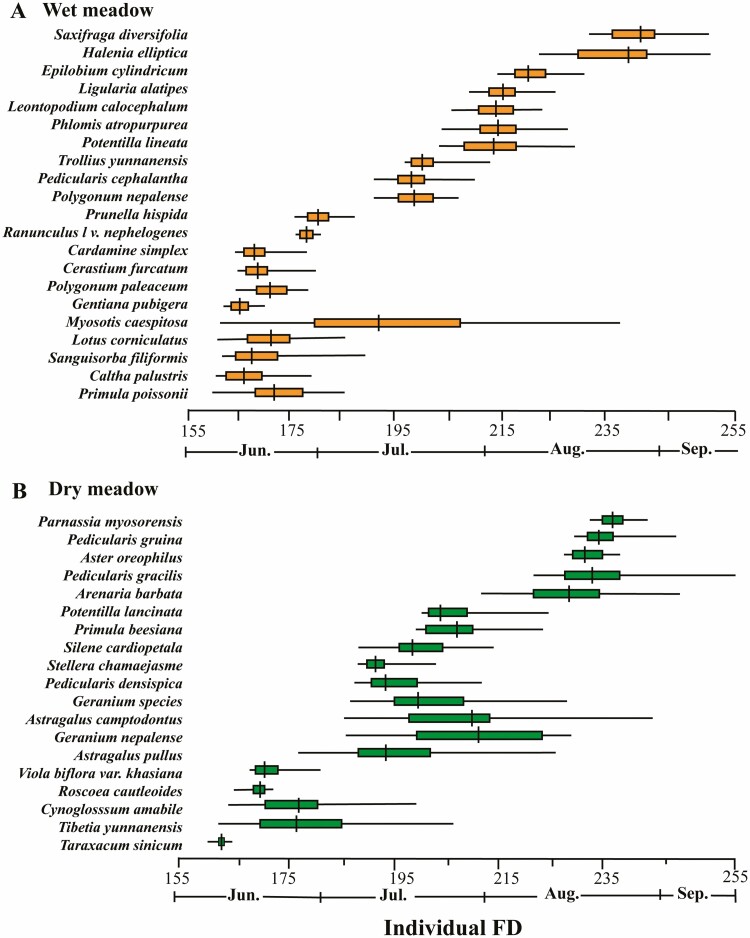
Individual FD (i.e. individual-level flowering duration among individuals) in two contrasting sub-alpine meadows (wet versus dry) on Yulong Mt., southwestern China. Shown are individual FDs for a (A) wet and (B) dry meadow. The solid thin line represents the entire flowering period of each species; the coloured bars indicate the peak flowering period (30–70% of flowers opened), and the vertical bar indicates the peak flowering day (50% of flowers opened). *N* = 41.

Individual FD varied widely among the studied species across the entire community, ranging from 3 days in *T. sinicum* to 75 days in *M. caespitosa* (Fig. 5A and B). Peak individual FD varied from 1 (*Ranunculus longicaulis* var*. nephelogenes*) to 26 days (*M*. *caespitosa*). In the wet meadow, FD for *M*. *caespitosa* was the longest (75 days), whereas *Ra. nephelogenes* (7 days) and *Gen. pubigera* (5 days) had the shortest FD. *Ajuga forrestii* (57 days) and *Astragalus camptodontus* (56 days) had the longest FD in the dry meadow, whereas *T. sinicum* (4 days) and *Roscoea cautleoides* (5 days) had a relatively short FD.

#### Floral longevity

**Figure 6.**
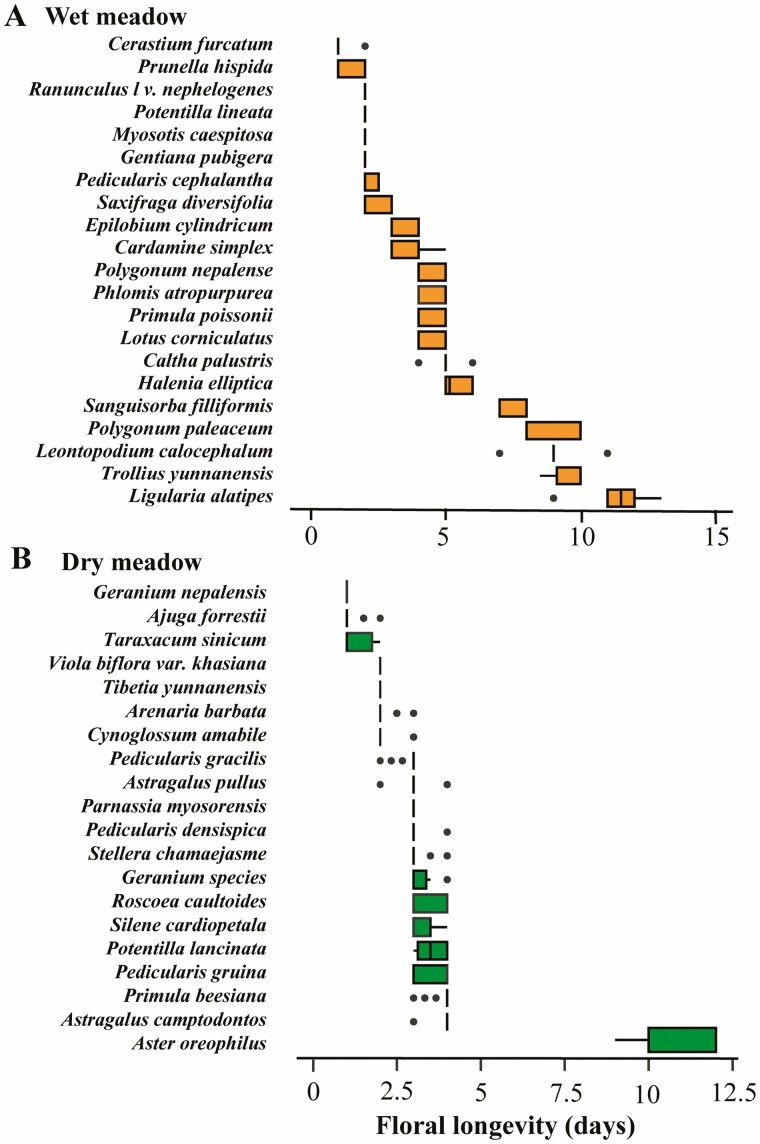
Floral longevity (i.e. flower-level longevity) in two contrasting sub-alpine meadows (wet versus dry) on Yulong Mt., southwestern China. Shown is floral longevity for a (A) wet and (B) dry meadow. Box plots with orange colour representing wet-meadow and green colour representing dry-meadow. 25th and 75th percentiles represent lower and upper box boundaries, respectively; the horizontal line inside the box represents the median. 10th and 90th percentiles are represented by the lower and upper error lines with filled circles data falling outside. The single vertical bar without a box represents those species that only flower for one day across all sampled flowers; *N* = 41.

Floral longevity varied between 1 day (*Geranium nepalense*) to 11 days (*Ligularia alatipes*) (Fig. 6A and B). In the wet meadow, *Li*. *alatipes* flowers remained open for 11 days on average, while floral longevity for *Cerastium furcatum* was one day. In the dry meadow, *Aste. oreophilus* floral longevity was the longest (11 days), whereas *Ger. nepalense* flowers lasted only a day.

### Floral traits and floral rewards in wet versus dry meadows

There was a high variation in pollen number, ovule number, and P/O per flower among the species studied in the entire community. Pollen number per flower varied widely between 418.8 ± 33.94 in *Pol. paleaceum* to 7525500 ± 259540.36 in *Cynoglossum amabile*. The number of ovules per flower ranged from one to 511 for *Pol. nepalense* and *Sa. Diversifolia*, respectively. P/O per flower varied between 59 in *Ger nepalense* and 1881375 in *Cy*. *amabile*. In the wet meadow, *Pol. paleaceum* (418.8 ± 33.94) and *M. caespitosa* (5199500 ± 173287.91) were the lowest and highest pollen-producing species, respectively. *Pol*. *paleaceum* had uni-ovulate ovules, and *Sa. diversifolia* had the highest number of ovules (511 ± 29.06) per flower. *Sa*. *diversifolia* had a low P/O (155), while *M*. *caespitosa* (1299875) had the highest. Further, in the dry meadow, *Ger. nepalense* (426 ± 34.32) and *Cy. amabile* (7525500 ± 259540.36) were the lowest and highest pollen-producing species, respectively. *Stellera chamaejasme* had uni-ovulate flowers, while *Pa. mysorensis* (182.6 ± 4.84) had the highest number of ovules per flower. P/O was lowest for *G*. *nepalense* (57.56) but highest for *Cy*. *amabile* (1881375) (see Supplementary Information).

The relationship between floral traits (pollen number, ovule number and P/O), population FD, individual FD and floral longevity varied significantly in the wet-, dry- and combined-meadow analyses (Table 1). In the wet meadow, individual FD and floral longevity showed a significant positive relationship with pollen number and P/O per flower, whereas no relationship with ovule production per flower was found. We also did not find any effects of population FD on each of the three floral traits. Further, only the floral longevity was significantly positively related to pollen number and P/O per flower in the dry meadow, as well as combined-meadow analysis (Table 1).

**Table 1.** Pollen and ovule number and P/O and their relation to population FD, individual FD, and floral longevity across two different habitats on Yulong Mt., southwestern China. Generalized Linear Model (GLM) with Quasi-Poisson distribution as the data were over-dispersed. Significance levels at < 0.05 are boldfaced. *N* = 37 (population FD) and 41 (individual FD and floral longevity).

Meadow community	Models	Variables	Estimate	*t*-Value	*P*-value
Wet	Population FD ~ pollen number + ovule number + P/O	Pollen number	0.00	1.87	0.08
		Ovule number	0.00	−1.68	0.12
		P/O	0.00	−1.74	0.11
	Individual FD ~ pollen number + ovule number + P/O	Pollen number	0.07	5.10	**<0.001**
		Ovule number	0.00	−0.16	0.87
		P/O	0.10	6.51	**<0.001**
	Floral longevity ~ pollen number + ovule number + P/O	Pollen number	0.00	2.18	**<0.05**
		Ovule number	0.00	−0.94	0.36
		P/O	0.00	2.21	**<0.05**
Dry	Population FD ~ pollen number + ovule number + P/O	Pollen number	0.00	0.38	0.71
		Ovule number	0.00	−1.06	0.32
		P/O	0.00	−0.37	0.72
	Individual FD ~ pollen number + ovule number + P/O	Pollen number	0.00	−0.74	0.47
		Ovule number	-0.01	−1.32	0.20
		P/O	0.00	0.74	0.47
	Floral longevity ~ pollen number + ovule number + P/O	Pollen number	0.00	2.00	**<0.05**
		Ovule number	0.00	−0.61	0.55
		P/O	0.00	2.03	**0.05**
Combined	Population FD ~ pollen number + ovule number + P/O	Pollen number	0.00	1.42	0.16
		Ovule number	0.00	−1.90	0.07
		P/O	0.00	−1.35	0.18
	Individual FD~ pollen number + ovule number + P/O	Pollen number	0.00	−0.64	0.53
		Ovule number	0.00	−1.14	0.26
		P/O	0.00	0.99	0.33
	Floral longevity ~ pollen number + ovule number + P/O	Pollen number	0.00	2.85	**<0.01**
		Ovule number	0.00	−0.81	0.42
		P/O	0.00	2.96	**<0.01**

The models with the low AIC values in floral rewards (nectar availability and pollen presentation) showed a positive effect on population FD (final AIC = 565.9, *z* = 81.27, *P* = <0.001, Table 2) but not for meadows. In contrast, individual FD and floral longevity did not show any response with floral rewards while having a significant positive response with the meadows (Individual FD: *z* = 45.65, *P* = <0.001 and floral longevity: *z* = 9.74, *P* = <0.001) (Table 2, Supporting Information—Figs. S1 and S2).

**Table 2.** The relation between flowering phenology and floral rewards across two habitats on Yulong Mt., southwestern China. Multiple regression GLM with Poisson distribution as the flowering phenology data were count data. Shown are the best-fit model with three sources of variation for each level of flowering phenology: (1) nectar: presence versus absence; (2) pollen presentation (Pp): open pollen presentation versus protected pollen presentation; (3) meadow: wet versus dry. Significance levels at <0.05 are boldfaced. *N* = 37 (population FD) and 41 (individual FD and floral longevity).

Variables	Model	Final AIC	Intercept	Nectar	Pp	Meadow	*z*-value	*P*-value
Population FD	Population FD ~ nectar + Pp	565.9	3.86	0.16	0.32	_	81.27	**<0.001**
Individual FD	Individual FD ~ meadow	Inf	3.03	−0.02	0.24	0.23	45.65	**<0.001**
Floral longevity	Floral longevity ~ meadow	Inf	1.50	0.07	−0.09	-0.36	9.74	**<0.001**

## Discussion

We conducted a community-wide comparison in flowering phenology at the population-, individual-, and flower levels across a wet versus dry habitat to investigate how patterns of flowering phenology can be explained by variation in water availability in each contrasting habitat. Further, by exploring different phenological levels and their effects on floral traits and rewards, we could more fully assess the maximum length of time male and female function may compensate for the potential costs associated with flower production and maintenance in a potentially dry environment. Our study provides one of the first community-wide assessments of potential sources of variation for flowering duration (population- and individual-level) and floral longevity among wet and dry habitats for a sub-alpine flora in the Hengduan Mountain region. Below, we discuss how this variation may impact flowering phenology in response to potential factors associated with increasingly drier habitats.

### Flowering phenology

Overall, we found a wide variation in the phenological patterns among the different species and meadows at our site. We observed a slight bimodal pattern in the number of flowering species, flowering individuals, and number of open flowers in the wet meadow with two peaks, followed by a sudden drop in values; the pattern was a more humped shape in the dry meadow (see Fig. 2). The bimodal pattern observed in the wet meadow could be because of early-season heavy rainfall that has happened for a short period (Nepal, personal observation), leading to an excess of water in the wet meadow, which may be unsuitable for early-season species that are typically better adapted to arid environments. Furthermore, the peak flowering time of mass flowering species (e.g. *Primula poissonii*) may have finished at the same time, indicating the start of other mass flowering species (e.g. *Phlomis atropurpurea* and *Li. alatipes*), suggesting a transition phase of the above mentioned flowering plant species as all three species have a higher abundance of individuals and flowers in the wet meadow.

In contrast, where the habitat does not hold water for a long duration (i.e. the dry habitat at our site), it may be favourable for a more uniform flowering period that is not impacted by sudden and short-term early-season rainfall. The hump-shaped pattern in the dry meadow might suggest that a dry environment is more suitable for a uniform flowering pattern that starts and ends at the specific time of the year (June–September) in the studied community. Our findings coincide with previous studies (i.e. Kudo and Suzuki, 1999), where a hump-shaped flowering pattern in sub-alpine/alpine plant communities was also found. Here, environmental factors in sub-alpine/alpine regions do not only affect population-level flowering phenology but also community-level flowering patterns (Kudo and Suzuki, 1999). Our results show that environmental influences on the pattern of flowering phenology can be substantial in sub-alpine habitats and should be monitored in future studies to fully understand changes in flowering phenology across multiple habitats.

Floral longevity was significantly longer in the wet meadow compared to the dry, whereas the opposite pattern was found in the dry environment for population and individual FD, which were significantly longer in the dry meadow compared to the wet. This result aligns with the phenological patterns described above and further suggests that flowers in dry habitats do not last long. Here, reducing pollinator rewards in dry habitats could impact successful fertilization events, where the wet meadow has enough water resources to provide continuous flower maintenance. However, this impact could also be negligible in the dry meadow, where longer population FD and individual FD could compensate for the resource cost associated with individual flowers. Previous studies suggest a similar pattern where the daily maintenance cost of flowers can increase with decreasing water supply, resulting in shorter floral longevity (Castro *et al.* 2008; Vandelook *et al.*, 2012; Jorgensen and Arathi, 2013; Ahmad *et al.*, 2021). However, as our study includes natural wet and dry habitats that are only 500 m apart where local rainfall conditions are essentially the same, it would appear that for flowering plant species better adapted to the dry habitats, future drought may have negligible effects on overall floral performance and reproductive output.

### Flowering phenology and its relation to pollen and ovule production

Both pollen number and P/O per flower showed a significant positive response on individual FD and floral longevity across independent wet and dry meadows and when combined, whereas the response was not significant for population FD in either habitat (Table 1). A strong positive response of individual FD and floral longevity on both pollen and P/O per flower coincides with earlier findings that show flowering phenology influences the number of pollen removed or received, which may, in turn, directly affect pollination and fertilization (Evanhoe and Galloway, 2002; Duan *et al.*, 2007). In addition, regardless of habitat, the number of pollen and P/O per flower had a strong positive response. In contrast, no such response was found for ovule number per flower across the different levels of flowering phenology in our study, also consistent with previous findings showing that variation in floral longevity may be more influenced by male fitness components than female (Ishii and Sakai, 2000; Gao *et al.*, 2015; Song *et al.*, 2022), although previous findings only occurred on a small scale (individual FD and floral longevity). Hence, our study uniquely suggests that each level of the phenological response to variation in pollen may be context-dependent, i.e. influenced by abiotic factors such as water availability, and as such, represents a significant source of variation rarely tested in the literature.

### Flowering phenology and its relation to floral rewards

In the current study, both nectar availability and pollen presentation differed significantly only for population FD, and the trend was consistent across both meadows. Further, pollen presentation differed significantly at the individual-level, a pattern that was also consistent across both meadows. These results firstly suggest that at the population-level, flowers providing nectar as rewards have shorter flowering duration than those that do not. In contrast, such a response was not evident at the individual- and flower-level. Secondly, flowers with enclosed pollen flower significantly longer at both the population- and individual-level than those with open pollen. For example, *Lo. corniculatus* in our study had pollen-enclosed flowers that flowered longer at the population level, whereas flowers of Asteraceae (*Le. calocephalum* and *Aste. oreophilus*) bloomed for a short duration. These results suggest that floral rewards at different levels of flowering phenology could be some of the few floral characteristics that can facilitate successful fertilization in fluctuating environments. While most studies on flowering phenology primarily focus on floral longevity (Stratton, 1989; Song *et al.*, 2022) and its response to variation in floral traits (Ashman and Schoen, 1994; Evanhoe and Galloway, 2002; Castro *et al.*, 2008), pollinator interaction (Bosch *et al.*, 1997; Cortés-Flores *et al.*, 2017), and abiotic factors related to unpredictable environments that occur in the sub-alpine region (Kudo and Hirao, 2006; Cortés-Flores *et al.*, 2017), to the best of our knowledge, no studies investigate the effects of floral rewards on population and individual FD in habitats that contrast in water availability.

## Conclusion

Where most previous research is limited to an individual flower’s longevity, our study offers a community-wide assessment providing fresh insights into additional potential sources of variation associated with flowering phenology at the population-, individual-, and flower-level and its response across (i) wet and dry habitats, (ii) pollen and ovule production per flower, and (iii) floral rewards (nectar availability and pollen presentation). Specifically, the significantly shorter duration of floral longevity in the dry habitat as compared to population and individual FD, which had an opposite response, suggests that water-dependent maintenance costs in flowering plants might play a key but hitherto hidden role in explaining the flowering phenology of sub-alpine plant species. In other words, where floral longevity may be impacted by a dry habitat, the effects on plant populations and individual plants might be negligible. Further, our results suggest that important functional floral traits (e.g. pollen number and P/O per flower) can be directly influenced by flowering phenology patterns at smaller scales and that flowering phenology patterns can significantly affect floral rewards. Future studies should more thoroughly investigate the impact of different wet and dry environments on a few focal species, as well as experimental studies with different levels of water availability, to investigate the adaptive response of specific flowering plant species to dry habitats.

## Supplementary Material

plae002_suppl_Supplementary_Data

plae002_suppl_Supplementary_Figures_S1

plae002_suppl_Supplementary_Figures_S2

## Data Availability

Excel data used for all the analysis is included as an Excel file in Supplementary Information.
